# Supplementary material for: PRC1 sustains the integrity of neural fate in the absence of PRC2 function
Source: eLife. 2022 Jan 7;11:e72769. doi: 10.7554/eLife.72769 (PMC8765755; doi:10.7554/eLife.72769)
Supplement: Transparent reporting form [file elife-72769-transrepform1.docx]

***eLife’s* transparent reporting form**

We encourage authors to provide detailed information *within their submission* to facilitate the interpretation and replication of experiments. Authors can upload supporting documentation to indicate the use of appropriate reporting guidelines for health-related research (see [EQUATOR Network](http://www.equator-network.org/%20)), life science research (see the [BioSharing Information Resource](https://biosharing.org/)), or the [ARRIVE guidelines](http://www.plosbiology.org/article/info:doi/10.1371/journal.pbio.1000412) for reporting work involving animal research. Where applicable, authors should refer to any relevant reporting standards documents in this form.

If you have any questions, please consult our Journal Policies and/or contact us: [editorial@elifesciences.org](mailto:editorial@elifesciences.org).

**Sample-size estimation**

- You should state whether an appropriate sample size was computed when the study was being designed
- You should state the statistical method of sample size computation and any required assumptions
- If no explicit power analysis was used, you should describe how you decided what sample (replicate) size (number) to use

Please outline where this information can be found within the submission (e.g., sections or figure legends), or explain why this information doesn’t apply to your submission:

Samples sizes were determined based on previous experience and published literature. No power analysis was employed however sample sizes were chosen using the same or comparable methods typically used in the field. Unless noted otherwise, all experiments were performed on at least three animals or biological samples of the same genotype. Designing our sample sizes according to published protocols and studies was done for consistency purposes as well as to allow comparison/verification of our quantifications to those found by others when applicable. Specific sample size information can be found in figures, figure legends and methods.

**Replicates**

- You should report how often each experiment was performed
- You should include a definition of biological versus technical replication
- The data obtained should be provided and sufficient information should be provided to indicate the number of independent biological and/or technical replicates
- If you encountered any outliers, you should describe how these were handled
- Criteria for exclusion/inclusion of data should be clearly stated
- High-throughput sequence data should be uploaded before submission, with a private link for reviewers provided (these are available from both GEO and ArrayExpress)

Please outline where this information can be found within the submission (e.g., sections or figure legends), or explain why this information doesn’t apply to your submission:

For each assay, independent experiments were repeated multiple times for all listed mouse genotypes, chick embryo manipulations, and motor neuron purifications to verify qualitative observations and for statistical analyses. Experiments were included in analysis if they met the same unbiased requirements applied across all genotypes. For example, experiments using chick neural tube electroporation were analyzed from samples of comparable efficiency (>30% MN labeled) to account for inherent method variability. Specific details for analysis of each experiment can be found in experimental procedures and specific sample sizes are reported in the results and figure legends. Results shown in figures are representative of results obtained from three or more animals unless otherwise noted.

**Statistical reporting**

- Statistical analysis methods should be described and justified
- Raw data should be presented in figures whenever informative to do so (typically when N per group is less than 10)
- For each experiment, you should identify the statistical tests used, exact values of N, definitions of center, methods of multiple test correction, and dispersion and precision measures (e.g., mean, median, SD, SEM, confidence intervals; and, for the major substantive results, a measure of effect size (e.g., Pearson's r, Cohen's d)
- Report exact p-values wherever possible alongside the summary statistics and 95% confidence intervals. These should be reported for all key questions and not only when the p-value is less than 0.05.

Please outline where this information can be found within the submission (e.g., sections or figure legends), or explain why this information doesn’t apply to your submission:

All experimental data are presented as biological replicates and were reproducible between animals and/or samples. When more informative, raw data was included in addition to other analyses. Statistical information including statistical test performed and exact p values are presented in the text of the results and methods as well as figure legends, when appropriate.

(For large datasets, or papers with a very large number of statistical tests, you may upload a single table file with tests, Ns, etc., with reference to sections in the manuscript.)

**Group allocation**

- Indicate how samples were allocated into experimental groups (in the case of clinical studies, please specify allocation to treatment method); if randomization was used, please also state if restricted randomization was applied
- Indicate if masking was used during group allocation, data collection and/or data analysis

Please outline where this information can be found within the submission (e.g., sections or figure legends), or explain why this information doesn’t apply to your submission:

Cohorts were grouped in results based on genotype. The experimental group was typically allocated as mutant versus control animals, by convention. Exact genotypes for mutant and control groups are defined in the main text and experimental procedures. For assays comparing control and mutant animals, experiments were performed on littermates comprising control and mutant animals and exact genotype of each animal was not known when experiments were done. Differences between animals of different genders are not expected, but were not formally tested.

**Additional data files (“source data”)**

- We encourage you to upload relevant additional data files, such as numerical data that are represented as a graph in a figure, or as a summary table
- Where provided, these should be in the most useful format, and they can be uploaded as “Source data” files linked to a main figure or table
- Include model definition files including the full list of parameters used
- Include code used for data analysis (e.g., R, MatLab)
- Avoid stating that data files are “available upon request”

Please indicate the figures or tables for which source data files have been provided:

Individual data points are shown in the main and supplemental figures, where appropriate. Details of software or programs used for data analyses are described in the experimental procedures section.
